# Identification of cell cycle as the critical pathway modulated by exosome-derived microRNAs in gallbladder carcinoma

**DOI:** 10.1007/s12032-021-01594-8

**Published:** 2021-10-16

**Authors:** Li Su, Jicheng Zhang, Xinglong Zhang, Lei Zheng, Zhifa Zhu

**Affiliations:** 1 Department of Integrated Traditional and Western Medicine in Oncology, The First Affiliated Hospital of Anhui Medical University, Hefei, 230022 People’s Republic of China; 2 Center of Integrated Traditional and Western Medicine in Oncology, Anhui Medical University, Hefei, 230022 People’s Republic of China; 3 Anhui University of Traditional Chinese Medicine, Hefei, 230012 People’s Republic of China

**Keywords:** Differentially expressed genes, Gallbladder cancer, miRNA, ceRNA, Carcinogenesis

## Abstract

Gallbladder cancer (GBC), the most common malignancy in the biliary tract, is highly lethal malignant due to seldomly specific symptoms in the early stage of GBC. This study aimed to identify exosome-derived miRNAs mediated competing endogenous RNAs (ceRNA) participant in GBC tumorigenesis. A total of 159 differentially expressed miRNAs (DEMs) was identified as exosome-derived miRNAs, contains 34 upregulated exo-DEMs and 125 downregulated exo-DEMs based on the expression profiles in GBC clinical samples downloaded from the Gene Expression Omnibus database with the R package. Among them, 2 up-regulated exo-DEMs, hsa-miR-125a-3p and hsa-miR-4647, and 5 down-regulated exo-DEMs, including hsa-miR-29c-5p, hsa-miR-145a-5p, hsa-miR-192-5p, hsa-miR-194-5p, and hsa-miR-338-3p, were associated with the survival of GBC patients. Results of the gene set enrichment analysis showed that the cell cycle-related pathways were activated in GBC tumor tissues, mainly including cell cycle, M phase, and cell cycle checkpoints. Furthermore, the dysregulated ceRNA network was constructed based on the lncRNA-miRNA-mRNA interactions using miRDB, TargetScan, miRTarBase, miRcode, and starBase v2.0., consisting of 27 lncRNAs, 6 prognostic exo-DEMs, and 176 mRNAs. Together with prognostic exo-DEMs, the STEAP3-AS1/hsa-miR-192-5p/MAD2L1 axis was identified, suggesting lncRNA STEAP3-AS1, might as a sponge of exosome-derived hsa-miR-192-5p, modulates cell cycle progression via affecting MAD2L1 expression in GBC tumorigenesis. In addition, the biological functions of genes in the ceRNA network were also annotated by Gene Ontology and Kyoto Encyclopedia of Genes and Genomes. Our study promotes exploration of the molecular mechanisms associated with tumorigenesis and provide potential targets for GBC diagnosis and treatment.

**Supplementary Information:**

The online version contains supplementary material available at 10.1007/s12032-021-01594-8.

## Background

Gallbladder carcinoma (GBC), the most common malignancy in the biliary tract, is highly lethal malignant [1]. Despite advances in multiple treatments including surgical resection, chemotherapy, and radiotherapy, more than 90% of patients are diagnosed at the advanced stage due to seldomly specific symptoms in the early stage of GBC [2–4]. These patients cannot be effectively treated and have an extremely low survival rate consequently [5]. Therefore, it is critical to focus on the exploration of new therapeutic interventions and early prognostic markers to improve the prognoses of patients with GBC.

The regulation of competing for endogenous RNAs (ceRNAs), which are transcripts that can regulate each other at the post-transcription level by competing for shared miRNAs, is involved in the physiology and development of diseases, especially cancer initiation and progression [6, 7]. MicroRNAs (miRNAs) are a class of small, endogenous, non-coding RNAs with a length of ∼ 22 nucleotides, playing an important role in regulating genes associated with malignant biological behavior in cancer cells [8–11]. The abnormal expression of long-chain non-coding RNA (lncRNA), which acts as microRNA decoys to modulate gene expression, is correlated with the occurrence and development of tumors and other diseases [12–14]. LncRNA could inhibit miRNA function, as “sponges” of natural miRNA, by competing with the binding of the microRNA response elements (MREs) in the complex [13, 15]. Some recent studies demonstrated that specific miRNAs are functionally involved in GBC development through modulating cell proliferation, apoptosis, migration, invasion, and metastasis, including miR-155, miR-200a, miR-182, miR-34a, and miR-130a [16–18]. Aberrant lncRNAs expression was indicative of the prognosis of GBC patients, and lncRNAs showed promise as diagnostic, prognostic, predictive biomarkers for GBC [19–21].

Exosomes, a type of extracellular vesicles (EVs) with diameters ranging from 40 to 100 nm, are widely released from many cell types into the extracellular space. Recently, mRNAs, lncRNAs, and miRNAs have been identified in exosomes. Dysregulation of exosome-derived ncRNAs is associated with tumor invasion, migration and metastasis, and drug resistance [22–24]. However, there are no studies specifically focused on the exosome-derived ncRNAs and their regulation of ceRNA networks in GBC. In the present study, we conducted a multi-step analysis using various R language packages on the expression profiles of clinical samples downloaded from the Gene Expression Omnibus (GEO) database to identify the differentially expressed mRNAs and ncRNAs in the GBC. The exosome-derived miRNAs associated with GBC patients’ survival were identified, and the related ceRNA network was established based on the lncRNA-miRNA-mRNA interaction. The gene set enrichment analysis (GSEA) of the differentially expressed genes regulated by exosome-derived miRNAs was identified to investigate the downstream regulation role of the exosome-derived miRNAs.

## Materials and methods

### Expression profiles information

The datasets GSE104165 and GSE74048 were downloaded from the GEO database (https://www.ncbi.nlm.nih.gov/geo/). The GSE104165 microarray expression profile dataset, which was based on GPL18402 ([Agilent-046064], Unrestricted_Human_miRNA_V19.0_Microarray), contained 40 GBC with long-term (*n* = 20) and short-term (*n* = 20) survival, and 8 normal gallbladder tissues [10]. The GSE74048 microarray expression profile dataset, which was based on GPL20115 ([Agilent-067406], Human CBC lncRNA + mRNA microarray V4.0), contained 3 pairs of human GBC tissues and the matched peri-carcinomatous tissues.

### Differential expression analysis

The differentially expressed genes (DEGs), lncRNAs (DELs), and miRNAs (DEMs) between GBC tissues and normal tissues of the GEO datasets, were calculated using the “limma” package with voom method in R, respectively [25]. The False Discovery Rate (FDR)—adjusted *P* value < 0.05 by the Benjamini–Hochberg method and |log2 fold change (FC)|> 2 were set as cut-off criteria for DEMs and DELs, while as adjusted *P* value < 0.05 and |log2FC|> 1.5 were for DEGs. Visualization of the identified DEGs and DEMs including volcano plot and heatmap were performed with the “ggplot2” and “pheatmap” packages of R, respectively [26]. Then, DEMs and exosome miRNAs, which were obtained from the exosome databases, ExoRBase and EVmiRNA, were intersected using the “VennDiagram” package of R, and the overlapped genes were identified as the exo-DEMs. The GBC tissues from GSE104165 were divided into the long survival group and short survival group based on the patients’ survival. Then, the expression profiles of exo-DEMs were analyzed between the two groups, and the survival-related exo-DEMs were selected for subsequent analysis.

### GSEA analysis of the survival-related exo-DEMs target genes

To elucidate the molecular mechanism of the exo-DEMs associated with survival, the interactions of miRNA-target genes were obtained from the reliable online miRNA-mRNA databases, including miRDB, TargetScan, and miRTarBase, using the “multiMiR” package in R (http://multimir.org/). Interactions of miRNA-target genes were obtained based on experimental verification of luciferase reporter assay. Then, the differential expression analysis of these target genes between GBC tissues and normal tissues from GSE74048 was performed, and the possible pathways and functions were predicted using the GSEA method with the Kyoto Encyclopedia of Genes and Genomes (KEGG) and Reactome databases. NOM *P* value < 0.05 was considered statistically significant. The results were visualized with the “clusterProfile” package in R [27].

### Construction of the survival-related exo-DEMs mediated ceRNA network

LncRNA-miRNA interaction pairs were predicted using the online databases, miRcode (http://www.mircode.org/index.php) and starBase v2.0 (http://starbase.sysu.edu.cn/starbase2/). Then integrated with the miRNA-mRNA interactions to establish a dysregulated lncRNA-miRNA-mRNA ceRNA network and visualized using Cytoscape software. Furthermore, the genes and the lncRNAs in ceRNA networks were intersected with DEGs and DELs, respectively, using Venn diagram, to identify the DEGs and DELs in the ceRNA networks. The gene expression was analyzed in GBC and normal tissue using the paired Student’s *t*-test. The two-sided *P* < 0.05 was considered statistically significant.

### Functional enrichment analysis and protein–protein interaction of the ceRNA network

To better understand the biological functions of the integrated ceRNA network, Gene Ontology (GO) covering biological processes (BP), molecular functions (MF), and cellular components (CC), enrichment analyses were performed. The whole human genome was set as the background, and functional categories with adjusted *P* < 0.01 were considered significant. The results were visualized with the “PerformanceAnalytics” (https://cran.r-project.org/web/packages/PerformanceAnalytics/) package of R. Furthermore, the protein interaction network was mapped with the Search Tool for the Retrieval of Interacting Genes/Proteins (STRING) website (http://string-db.org/) and visualized using Cytoscape software [28].

## Results

### Identification of DEMs, DELs, and DEGs in gallbladder carcinoma

**Fig. 1:**
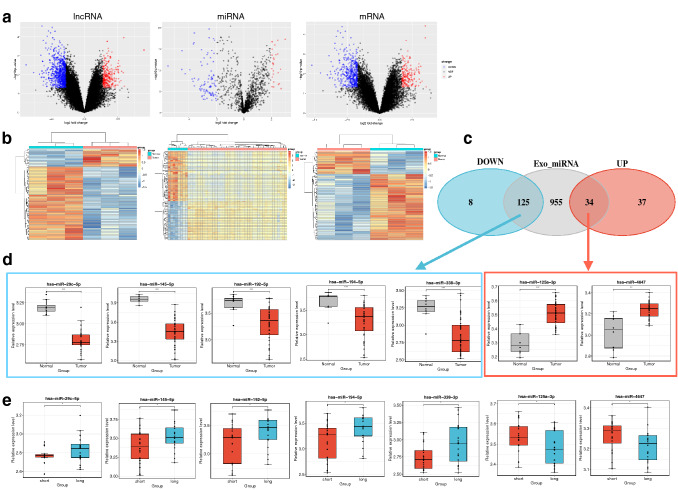
Identification of DEL, exosomal DEMs and DEGs between GBC and normal tissues. **a** Volcano plot of DEL, DEMs, and DEGs for datasets from GEO. X-axis: log2 fold change; Y-axis: − log 10 (*P* value) for each gene; vertical-dotted lines: fold change ≥ 2 or ≤ 2 for DELs and DEMs, fold change ≥ 1.5 or ≤ 1.5 for DEGs; horizontal-dotted line: the significance cut off (adjusted *P* value = 0.05). The red dot represents up-regulated genes, and the blue dot represents down-regulated genes. **b** Gene expression heatmap of DEL, DEMs, and DEGs, respectively. **c** Venn diagrams of the overlapping genes between exosome-derived miRNAs with up-regulated DEMs and down-regulated DEMs, respectively. **d** Box plots that represent the expression levels of exo-DEMs between tumor tissues and normal tissues. **e** Box plots that represent the expression levels of exo-DEMs between the tissues from long survival and short survival patients. **** indicates *P* < 0.0001, *** indicates *P* < 0.001, ** indicates *P* < 0.01

A total of 843 DEGs and 895 DELs between GBC tissues and the matched peri-carcinomatous tissues were obtained after analyzing expression profiles from GSE74048 datasets. Among them, 246 mRNAs and 716 lncRNAs were significantly upregulated; while 597 mRNAs and 179 lncRNAs were significantly downregulated, respectively. Meanwhile, 204 DEMs, including 71 up-regulated and 133 down-regulated miRNAs, between GBC tissues and normal tissues were obtained from GSE104165 dataset, respectively. The volcano plots and heatmaps illustrated the significant differences and distribution of the fold change in DEMs, DELs, and DEGs (Fig. 1a, b).

### The exosome DEMs associated with the GBC patients’ survival

To screen the exosome-related DEMs in GBC compared with normal tissues, Venn diagram analysis was used to obtain the intersection between DEMs and miRNAs from exosomes. Subsequently, a total of 159 overlapped DEMs were identified, containing 34 upregulated exo-DEMs and 125 downregulated exo-DEMs (Fig. 1c). Seven survival associated exo-DEMs were obtained through the differential expression analysis between the long survival group and the short survival group (Fig. 1d), including 2 up-regulated exo-DEMs, hsa-miR-125a-3p and hsa-miR-4647, and 5 down-regulated exo-DEMs, hsa-miR-29c-5p, hsa-miR-145a-5p, hsa-miR-192-5p, hsa-miR-194-5p, and hsa-miR-338-3p. Results showed that the 2 up-regulated exo-DEMs were up-regulated in the short survival group, while the 5 down-regulated exo-DEMs were up-regulated in the long survival group (Fig. 1e).

### GSEA analysis of the survival-related exo-DEMs target genes

**Fig. 2:**
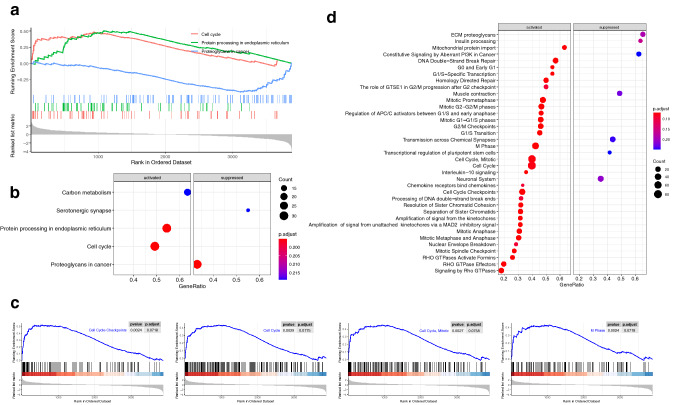
GSEA analysis revealed the target genes of seven survival-related exo-DEMs mainly enriched in cell cycle-related pathways. **a**, **b** Illustrate the top elements significantly enriched in the KEGG pathway with a *P* value of less than 0.05. **c**, **d** The Reactome pathway enriched in up-regulated target genes and down-regulated target genes with a *P* value of less than 0.01, respectively

To elucidate the molecular mechanism of the exo-DEMs with patients’ survival, GSEA analysis of target genes was performed. Result showed that the GBC tumor tissues were mainly enriched in KEGG pathways including cell cycle (hsa04110, *P* = 0.002), protein processing in endoplasmic reticulum (hsa04141, *P* = 0.002), and carbon metabolism (hsa01200, *P* = 0.004), while as the normal tissues were mainly enriched in proteoglycans in cancer (hsa05205, *P* = 0.001), and serotonergic synapse (hsa04726, *P* = 0.004) (Fig. 2a; SI 1). That indicated the genes in cell cycle, protein processing in endoplasmic reticulum, and carbon metabolism were activated in GBC tumor tissues, and the genes in pathways of proteoglycans in cancer, and serotonergic synapse were suppressed (Fig. 2b). Reactome pathway enrichment also indicated that the cell cycle-related pathways were activated in GBC tumor tissues, including cell cycle (R-HSA-1640170, *P* = 0.002), cell cycle, mitotic (R-HSA-69278, *P* = 0.003), M phase (R-HSA-68886, *P* = 0.002), cell cycle checkpoints (R-HSA-69620, *P* = 0.002) (Fig. 2c, d; SI 2).

### Survival-related exo-DEMs mediated ceRNA network

**Fig. 3:**
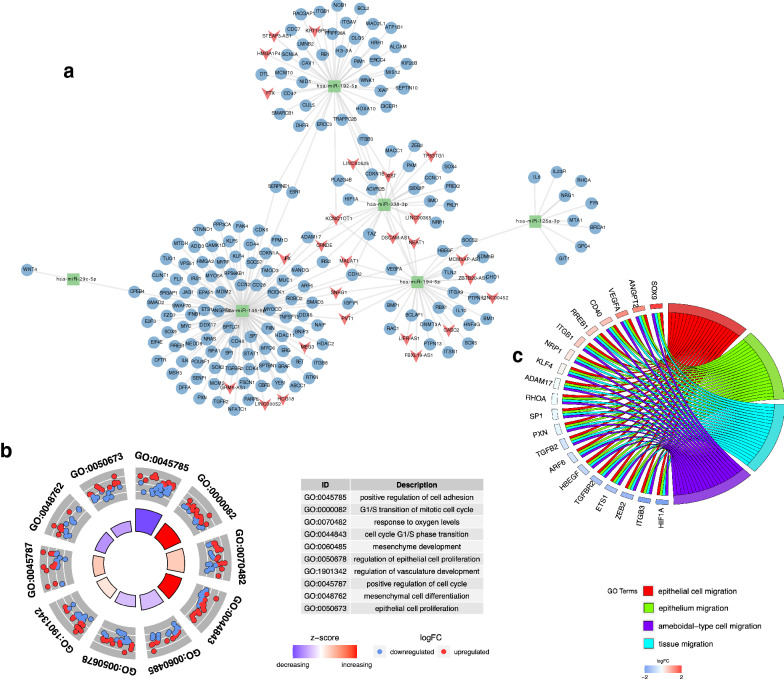
The ceRNA network of survival-related exo-DEMs, lncRNAs, and mRNAs. **a** Green rectangles indicate exo-DEMs, red arrows indicate lncRNAs, and blue circles indicate mRNAs; **b** illustrate the top 10 elements significantly enriched in the three GO categories; **c** the significant GO terms associated with the DEGs*. P* value is less than 0.01

To better understand the biological regulation roles of the exo-DEMs with patients’ survival, we constructed the dysregulated ceRNA network based on the lncRNA-miRNA-mRNA interactions. The regulatory relationship between 176 mRNAs and 6 of survival-related exo-DEMs with patients’ survival was found based on experimental verification of luciferase reporter assay (SI 3). Then, 27 lncRNAs were predicted to interact with the exo-DEMs using the online databases, miRcode, and starBase v2.0. Finally, integrated the relationship of miRNAs and mRNAs, miRNAs and lncRNAs, a ceRNA network consisting of 27 lncRNAs, 6 miRNAs, and 176 mRNAs was constructed (Fig. 3a). In addition, we calculated the connection degree of each gene by topology to clarify its importance in the ceRNA network. mRNA (CDH2), lncRNA (KCNQ1OT1), and miRNAs (hsa-miR-145-5p) were considered the most important genes among the lncRNAs, miRNAs, and mRNAs, respectively (SI 4).

### Functional enrichment and expression analysis of genes in the ceRNA network

GO enrichment and KEGG pathway analysis were used to investigate the mechanisms associated with the ceRNA network with the threshold set as adjusted *P* < 0.01. The significantly enriched GO terms were illustrated and available in Fig. 3b, c and SI 5. Most importantly, among the enriched biological processes were mainly including cellular process (GO:0009987, *P* = 2.96E-17), biological regulation (GO:0065007, *P* = 8.64E-22), response to stimulus (GO:0050896, *P* = 5.94E-24). Among the molecular functions obtained from GO enrichment analysis were organic cyclic compound binding (GO:0097159, *P* = 7.62E-10), protein binding (GO:0005515, *P* = 3.22E-29), heterocyclic compound binding (GO:1901363, *P* = 8.71E-10). The most notable significantly enriched cellular components included cell (GO:0005623, *P* = 4.94E-10), intracellular (GO:0005622, *P* = 5.57E-11), intracellular organelle (GO:0043229, *P* = 7.91E-10). Meanwhile, KEGG analysis suggested that the significantly enriched pathways including Pathways in cancer (hsa05200, *P* = 3.48E-24), Proteoglycans in cancer (hsa05205, *P* = 2.05E-21), MicroRNAs in cancer (hsa05206, *P* = 5.13E-24), Cell cycle (hsa04110, *P* = 1.05E-13), Hepatocellular carcinoma (hsa05225, *P* = 4.74E-13) (SI 6).

**Fig. 4:**
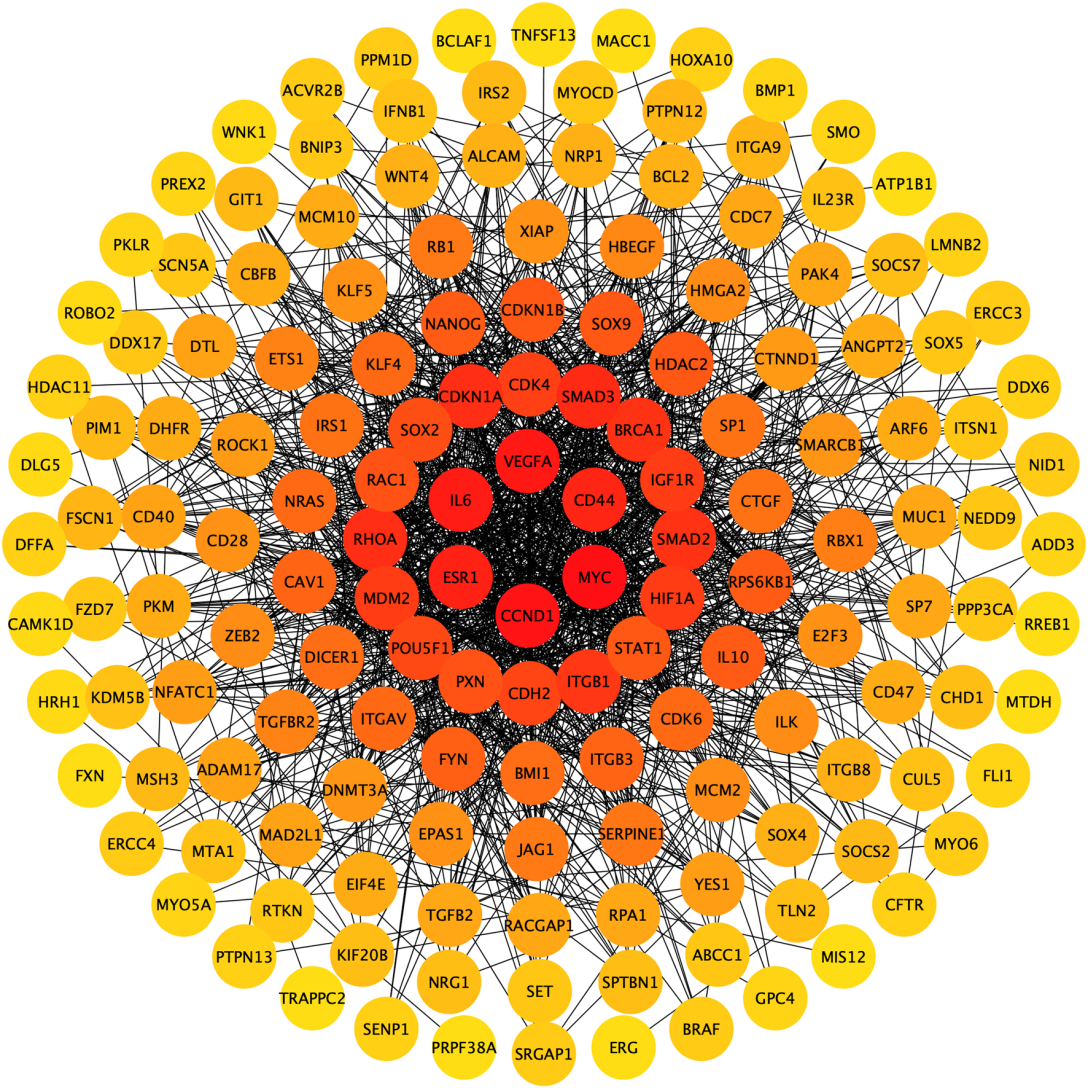
Protein–protein interaction network displaying the interactions among the genes in the ceRNA networks, as determined with STRING and illustrated in Cytoscape

**Fig. 5:**
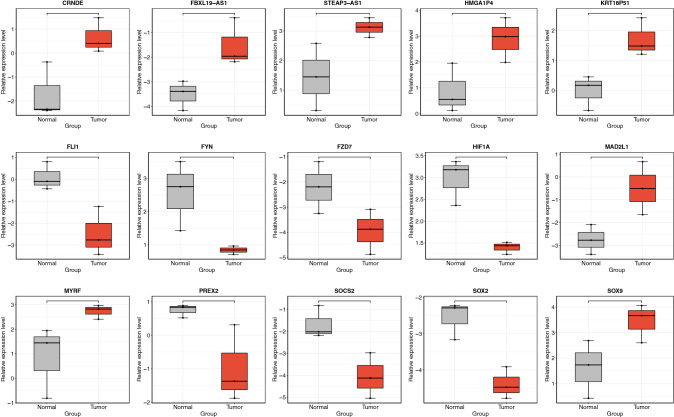
Box plots that represent the differentially expression of mRNAs and lncRNAs in the ceRNA networks between tumor tissues and normal tissues. **** indicates *P* < 0.0001, *** indicates *P* < 0.001, ** indicates *P* < 0.01

Protein–protein interaction of the gene in the ceRNA networks shown that the high-scoring proteins were obtained including CD44, MYC, CCND1, ESR1, IL6, and VEGFA, which suggesting that they might jointly regulate the occurrence and development of tumor (Fig. 4). Expression analysis indicated that five lncRNAs and ten mRNAs were differentially expressed between GBC tumor tissues and normal tissues, including seven mRNAs down-regulated in tumor tissues, while the other three mRNAs and all the five lncRNAs up-regulated in tumor tissues (Fig. 5).

### Cell cycle as a critical pathway in GBC tumorigenesis

**Fig. 6:**
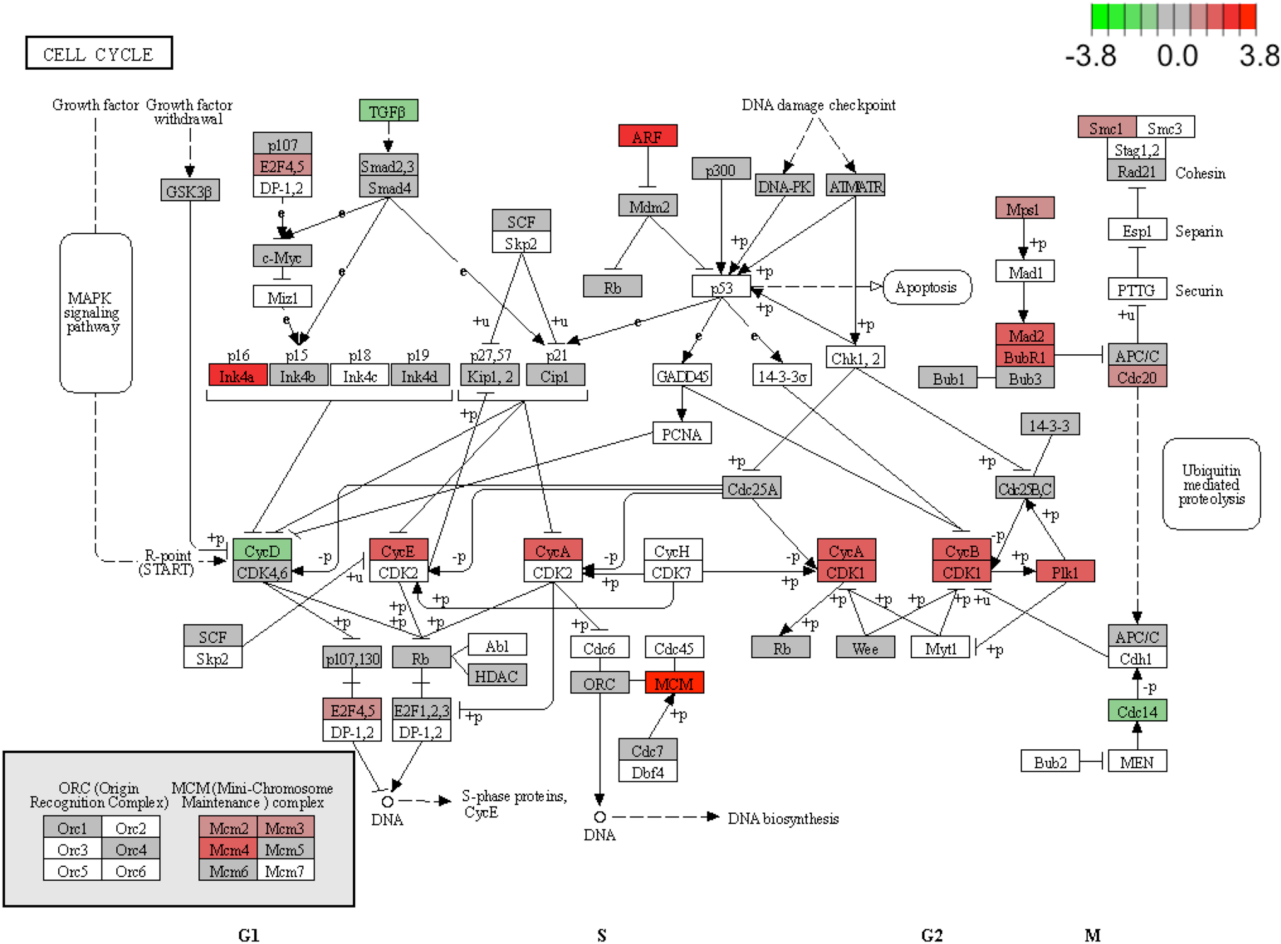
Differential expression of genes associated in the cell cycle pathway between tumor and normal tissues. The red color represents up-regulated genes, the green color represents down-regulated genes, and the gray color represents non-significantly expressed genes

Based on the integrated ceRNA network and GSEA analysis, the STEAP3-AS1/hsa-miR-192-5p/MAD2L1 axis, which participants in cell cycle pathway, was identified as critical in GBC tumorigenesis. Besides, as shown in Fig. 6, we also found that mainly members in this pathway were differentially expressed, including 17 genes were up-regulated in GBC tumor tissues, while three genes were down-regulated.

## Discussion

As dysregulation of exosomal ncRNAs has been highlighted for critical functions in tumor invasion, migration and metastasis, and drug resistance. With the purpose of identifying the exosomal miRNAs mediated ceRNA participant in GBC tumorigenesis and to investigate the potential biomarkers for better detection and therapy, we analyzed the gene expression profiles of the GEO datasets and established a lncRNA-miRNA-mRNA network mediated by prognostic exo-DEMs at the transcriptome-wide level to provide a useful foundation for the regulatory function research in GBC.

We identified 204 DEMs, including 71 up-regulated and 133 down-regulated miRNAs, between GBC tissues and normal tissues. A total of 159 DEMs was identified as exosomal miRNAs, containing 34 up-regulated exo-DEMs and 125 down-regulated exo-DEMs. Seven of these exo-DEMs were differentially expressed between the long-term survival group and short-term survival group of GBC patients, including 2 up-regulated hsa-miR-125a-3p and hsa-miR-4647, and 5 down-regulated hsa-miR-29c-5p, hsa-miR-145a-5p, hsa-miR-192-5p, hsa-miR-194-5p, and hsa-miR-338-3p, indicating that they had a prognostic relationship with the survival of GBC patients. Similar results were showed in other studies in different kinds of cancers. Overexpression of hsa‑miR‑125a‑3p significantly inhibited the proliferation and invasion of lung cancer cells, and also is readily accessible as a diagnostic biomarker for early-stage colon cancer (CRC) [29, 30]. Exosomes secreted by human adipose-derived MSCs (adMSC-Exo) can transfer miR-125a to endothelial cells and promote angiogenesis through repressing the angiogenic inhibitor delta-like 4 (DLL4) by targeting its 3′ untranslated region [31]. miR-192-5p, which is associated with TRIM44, its upregulation suppressed tumor behaviors in lung cancer cells [32]. Bone marrow-derived mesenchymal stem cells (BMSCs)-secreted miR-192-5p can delay the event of the inflammatory response in rheumatoid arthritis (RA) [33]. Researchers also found that human adipose mesenchymal stem cell exosomal miR-192-5p targeted IL-17RA to regulate Smad pathway in hypertrophic scar (HS) fibrosis [34]. Meanwhile, in contrast to our results, miR-192-5p was up-regulated in nasopharyngeal carcinoma (NPC) tissues and was identified as “Exosomal onco-miRs” in esophageal cancer (EC). Exosomal miR-194-5p enhanced DNA damage response in the residual tumor repopulating cells to potentiate tumor repopulation [35, 36]. miR-194-5p was also found significantly downregulated in exosomes of chronic kidney disease (CKD) patients serum [37, 38].

After that, GSEA analysis was performed to investigate the molecular mechanism of the prognostic exo-DEMs. Results indicated that the enriched KEGG and Reactome pathways which activated in GBC tumor tissues were mainly associated with the cell cycle-related pathways including cell cycle, M phase, and cell cycle checkpoints. Furthermore, the dysregulated ceRNA network based on the lncRNA-miRNA-mRNA interactions was constructed, consisting of 27 lncRNAs, 6 prognostic exo-DEMs, and 176 mRNAs. GO enrichment and KEGG pathway analysis also showed that the genes of the ceRNA network were mainly enriched in cell cycle-related GO terms and KEGG pathways. Before the reproductive cycle of replicating DNA, cells enter a phase called G1 during which they release plenty of signals that contribute to cell division and cell fate. miRNAs regulate genes involved in checkpoints, DNA repair, and cell cycle. Dysregulated miRNAs can lead to the loss of cell cycle regulation and finally contribute to pathological situations, such as cancer [38–40]. Expression analysis indicated that 5 lncRNAs and 10 mRNAs in the ceRNA network were differentially expressed between GBC tumor tissues and normal tissues. Together with prognostic exo-DEMs, the STEAP3-AS1/hsa-miR-192-5p/MAD2L1 axis was identified. Due to the main members in the cell cycle pathway were differentially expressed including MAD2L1, we speculated that the STEAP3-AS1/hsa-miR-192-5p/MAD2L1 axis was critical in GBC tumorigenesis through regulation of cell cycle. A recent study has found that STEAP3-AS1 downregulation could increase the expression of cyclin-dependent kinase inhibitor 1C (CDKN1C) by STEAP3 upregulation and modulate the cell cycle progression in CRC [41]. STEAP3-AS1 was also identified as a novel, potential prognostic biomarker, and therapeutic target for tongue squamous cell carcinoma (TSCC) [42]. STEAP3-AS1, together with HOTAIR and SOX21-AS1, as the exo-lncRNA signature, was proven to be an independent prognostic factor in glioblastoma (GBM) [43]. MAD2L1, MAD2 mitotic arrest deficient-like 1, is a critical mitotic checkpoint gene. Overexpressing MAD2L1 has shown the effect on cancer cell proliferation, migration, invasion, and cell cycle arrest [44–46]. Activation of miR-192/215 by p53 includes a set of genes which regulate DNA synthesis and G1 and G2 checkpoints, including CDC7, MAD2L1, and CUL5, to induce cell arrest and reduce tumor cell growth [47–50]. In our study, we found that STEAP3-AS1 and MAD2L1 were up-regulated in GBC tumor tissues, while as hsa-miR-192-5p was down-regulated, suggesting lncRNA STEAP3-AS1, might as a sponge of exosome-derived hsa-miR-192-5p, modulates cell cycle progression via affecting MAD2L1 expression in GBC tumorigenesis. Further experimental studies are required to verify this hypothesis. These findings suggest that the ceRNA network have significant effects on the pathogenesis and prognosis of GBC.

## Conclusion

This study provides a better understanding of the lncRNA-miRNA-mRNA regulatory mechanism in GBC mediated by exosome-derived miRNAs. In future research, we will explore the prognostic genes and their potential function of the ceRNA axis based on the present study.

## Supplementary Information

Below is the link to the electronic supplementary material.

Supplementary file1 (XLSX 11 kb)

Supplementary file2 (XLSX 20 kb)

Supplementary file3 (XLSX 26 kb)

Supplementary file4 (XLSX 17 kb)

Supplementary file5 (XLSX 182 kb)

Supplementary file6 (XLSX 20 kb)

## Data Availability

GEO belongs to public database. The patients involved in the database have obtained ethical approval. Users can download relevant data for free for research and publish relevant articles. Our study is based on open-source data, so there are no ethical issues and other conflicts of interest.
